# Mycorrhizal legacy mediates seedling success following timber harvesting in Northeastern forests

**DOI:** 10.1002/eap.70281

**Published:** 2026-07-08

**Authors:** Amelia Fitch, Sarah Goldsmith, Anthony W. D'Amato, Eva O. L. Legge, Audrey Adamchak, Dustin Gannon, Alexandra M. Kosiba, Kevin Evans, Caitlin Hicks Pries

**Affiliations:** ^1^ Department of Biological Sciences Dartmouth College Hanover New Hampshire USA; ^2^ Ecology, Evolution, Environment, and Society Program Dartmouth College Hanover New Hampshire USA; ^3^ Rubenstein School of Environment and Natural Resources University of Vermont Burlington Vermont USA; ^4^ Department of Biology Syracuse University Syracuse New York USA; ^5^ Forest Ecosystems and Society College of Forestry, Oregon State University Corvallis Oregon USA; ^6^ Extension University of Vermont S. Burlington Vermont USA; ^7^ Dartmouth College Woodlands Office Milan New Hampshire USA; ^8^ Present address: Forest Ecosystems and Society College of Forestry, Oregon State University Corvallis Oregon USA

**Keywords:** common mycorrhizal network, mycorrhizal fungi, seedling survival, soil legacy effects, soil nitrogen, timber harvesting

## Abstract

Overstory trees rely on arbuscular mycorrhizal (AM) or ectomycorrhizal (EcM) fungi for establishment and growth. However, we know very little about how timber harvesting affects the biotic (e.g., mycorrhizal fungi) and abiotic (e.g., nutrient availability) legacies left behind by AM and EcM trees. These belowground mycorrhizal legacies, including potential shifts in abundance of mycorrhizal fungi, may differentially affect seedling success. To address this knowledge gap, we investigated how the legacy of AM and EcM forest stands affected seedling survival and growth, soil fungal communities, and soil chemistry including nutrient availability and pH following timber harvesting in a temperate hardwood forest. We established 16 plots, half of which were AM‐ and half of which were EcM‐dominated. Eight of the 16 plots were harvested and then planted with four AM‐ and four EcM‐associated seedling species in a split‐plot design. The eight unharvested control plots allowed us to compare changes in fungal biomass, and soil pH and nutrients in the harvested plots to a baseline. AM fungal biomass decreased in both AM and EcM plots only in the first fall after timber harvesting, while EcM fungal biomass continued to decrease in both AM and EcM plots over three growing seasons. The mycorrhizal legacy also affected seedling success; AM seedlings had higher survival when planted in AM legacy plots, but there was no effect on EcM seedlings. AM and EcM seedling growth was unaffected by legacy. Patterns in foliar nitrogen (N) showed that although AM seedlings closer to plot edges may have acquired more N through mycorrhizal fungi relative to EcM seedlings at plot edges, they also had lower percent N, indicating a tradeoff between increased access to mycorrhizal networks and competition with proximity to live roots. Alternatively, this mycorrhizal legacy effect could have been driven by preexisting soil characteristics. Given that some AM tree species are sensitive to soil calcium (Ca), higher soil extractable Ca in AM legacy plots suggests a complex set of mechanisms driving seedling success beyond potential access to mycorrhizal fungi.

## INTRODUCTION

Forest regeneration in North America can drive ecosystem services such as carbon storage and nutrient cycling (Dean et al., 2017; Marshall, 2000). These services may be under threat as seedling recruitment faces increasing stress under a warming climate, shifting precipitation regimes, and potential threats from invasive pests (Beckage et al., 2008; Cleavitt et al., 2018; Fischer et al., 2013). Belowground legacies, including microbial communities and soil abiotic characteristics, could potentially increase seedling success and forest regeneration rates. While biotic facilitative effects from mycorrhizal fungi can increase seedling survival (Averill et al., 2022), abiotic soil conditions such as soil nutrient content can also drive seedling performance (Dhiedt et al., 2024). However, in managed forest landscapes of North America, it is not yet clear how belowground legacies left behind by harvested trees will affect seedling success. Furthermore, overstory tree species may leave behind different biotic and abiotic legacies depending on their mycorrhizal association, either arbuscular mycorrhizal (AM) or ectomycorrhizal (EcM). Consequently, investigating the effects of tree‐mycorrhizal legacies on seedling success following timber harvests is critical to promoting forest resilience and adaptive silviculture under global change (D'Amato et al., 2023; Ellison et al., 2005; Wikle et al., 2024).

Timber harvesting can cause rapid shifts in soil nutrient cycling and availability to affect seedling survival and forest regeneration (Caihong et al., 2023; Elser et al., 2007; Nelson et al., 2024). Within the first few years following harvesting, elevated amounts of decaying twigs, leaves, and roots can increase soil nutrient availability (Covington, 1981; Dahlgren & Driscoll, 1994). This short‐term flush of nutrients, especially inorganic nitrogen (N), provides regenerating seedlings with resources needed to establish and grow (Timmer, 1997). However, in temperate forest ecosystems, AM seedlings may be better able to capitalize on elevated soil N availability following harvesting in comparison to EcM seedlings.

Patterns of N cycling in AM‐dominated soils suggest that AM trees may be better adapted to systems with higher nutrient content. The higher N content in AM tree litter helps maintain faster cycling of inorganic N in soils beneath adult AM trees compared to EcM trees, where most of the N is held in soil organic matter (SOM; Phillips et al., 2013; Sun et al., 2018). AM fungi are also generally more efficient at inorganic N uptake than EcM fungi (Liese et al., 2018), which can promote faster AM tree growth relative to EcM trees in N‐rich soils (DeForest & Snell, 2020). This difference in adaptation to soil N conditions may explain how, over the last three decades in temperate ecosystems, AM trees have outcompeted EcM trees under anthropogenic N deposition across much of the eastern United States (Jo et al., 2019). Although these patterns suggest that AM seedlings could have a growth advantage over EcM seedlings due to SOM decomposition and resulting flush of inorganic soil N following timber harvesting, lingering biotic legacy effects of soil pathogens may simultaneously act to reduce AM seedling survival (Rotter et al., 2020; Sheldrake et al., 2018).

Timber harvesting will likely decrease mycorrhizal biomass, reducing potential benefits of a mycorrhizal legacy, while soil pathogens may persist and could reduce initial seedling survival (Borgmann‐Winter et al., 2022; Magee et al., 2025). Relative to EcM seedlings, AM seedlings often have lower survival when planted near live conspecific adult trees due to an elevated abundance of host‐specific soil pathogens (Bennett et al., 2017; Jiang et al., 2020; Liang et al., 2020, 2021), whereas EcM seedlings often benefit from a conspecific legacy (Delavaux et al., 2023). However, AM and EcM seedlings do not always follow this pattern. Field studies in temperate forests have found that EcM seedlings suffered negative effects near conspecific adults (Jevon et al., 2020, 2022; Jia et al., 2020), and some AM forests may be maintained through positive legacy feedbacks (Averill et al., 2022; Tourville et al., 2024). However, the strength of these biotic legacy effects on seedling survival may depend on the environmental conditions, such as soil nutrient and water content (Neat et al., 2025). Thus, postharvest biological legacy effects may depend on other abiotic factors, such as nutrient availability, for seedlings planted in soils with different mycorrhizal legacies.

An alternative mechanism to these biotic legacies of pathogens and mycorrhizal facilitation is that tree distributions between mycorrhizal types (MTs) may reflect adaptation to abiotic legacies with soil characteristics that maintain mycorrhizal dominance in forests (Averill et al., 2022). Decades of ecological research have shown that tree species tend to dominate in soils that align with their physiological tolerances and nutrient demands (John et al., 2007; Piedallu et al., 2016; Walthert & Meier, 2017; Zukswert et al., 2025). In this view, differences in seedling regeneration could be affected not only by biotic interactions, but also by persistent differences in soil chemistry shaped by edaphic characteristics. For example, trace minerals necessary for plant functions, such as calcium (Ca), can vary considerably with bedrock type and weathering rates (Hynicka et al., 2016), and tree species can reinforce these gradients in mineral nutrient availability through differences in litter quality (Phillips et al., 2013). Globally, EcM trees tend to grow in more acidic soils with lower concentrations of base cations like Ca than AM trees (Cleavitt et al., 2018; Lin et al., 2022; Tourville et al., 2024). As such, systematic differences in soil abiotic properties like Ca availability between AM‐ and EcM‐dominated forests may also contribute to differences in seedling success following timber harvesting.

With the potential for soil biotic and abiotic legacies to interactively affect seedling success, it is critical to understand the belowground mechanisms that affect the survival and growth of AM and EcM seedlings following timber harvests. Such insight could help forest managers increase forest resilience and productivity in the face of global change stressors. To address this knowledge gap, we sought to (1) examine how seedling survival and growth varied depending on the mycorrhizal associations of recently harvested mature trees, hereafter referred to as the mycorrhizal legacy, and (2) investigate how timber harvesting may have affected soil nutrient availability and fungal communities. Given previous research showing that increased access to EcM fungi can offset the negative effects of soil pathogens (Jiang et al., 2020) relative to AM fungi (Bennett et al., 2017), EcM‐associated seedlings may have higher survival in soils previously dominated by EcM trees, whereas AM seedlings could experience reduced survival when planted in AM legacy soils. Alternatively, because AM trees and associated fungi may be better equipped to capitalize on greater soil inorganic N (Liese et al., 2018) and are sensitive to soil Ca content (Cleavitt et al., 2018), AM‐associated seedlings may experience an early growth advantage when planted in AM legacy soils due to underlying soil nutrient conditions.

## METHODS

### Site description and experimental setup

Our study took place in the Clement Woodlot, a northern mixed‐deciduous forest in Corinth, Vermont (44°02′48.4″ N, 72°19′03.0″ W), dominated by overstory tree species sugar maple (*Acer saccharum*) and white ash (*Fraxinus americana*) across AM‐dominated areas, and by eastern hemlock (*Tsuga canadensis*), yellow birch (*Betula alleghaniensis*), and American beech (*Fagus grandifolia*) in EcM‐dominated areas. Corinth experiences a mean annual temperature and total annual precipitation of 5.3°C and 1139 mm, respectively (PRISM Climate Group, 2014).

**FIGURE 1:**
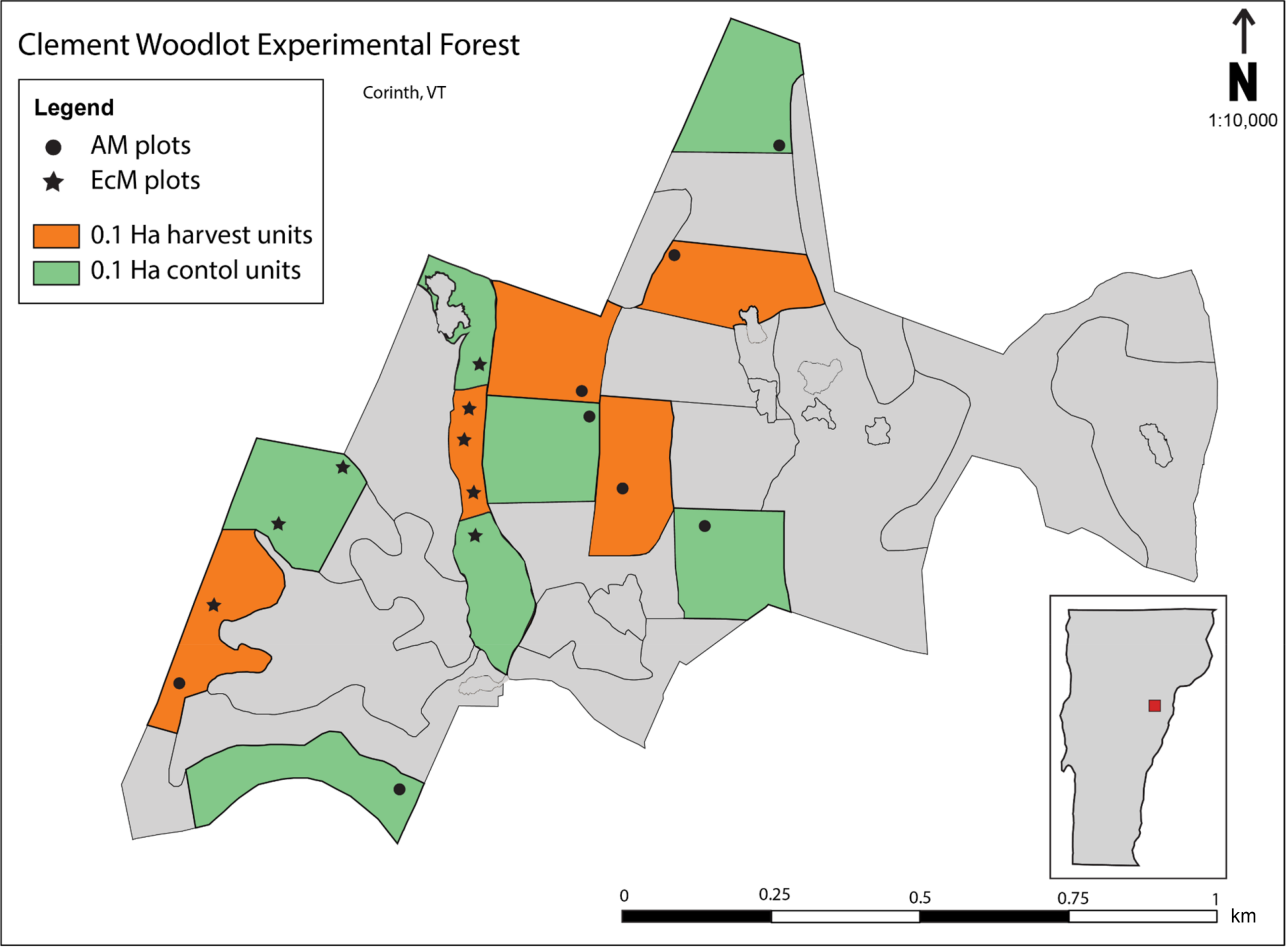
Map of Corinth, VT, research site, where colored sections indicate areas containing 0.1 ha plots (black dots) that were treated as control (green) or gap cuts (orange). Points within units indicate plot locations, where star shaped points were dominated by ectomycorrhizal (EcM)‐associating trees and circle shaped points were dominated by arbuscular mycorrhizal (AM)‐associating trees. Edited from base map created by Riley Patry, Wildlife Ecologist at Dartmouth College Woodlands.

We established 16 0.1‐ha plots where half of the plots were dominated (>80% basal area) by EcM trees and half by AM trees (Figure 1). To determine plot mycorrhizal dominance, we measured the dbh for all trees that were >10 cm in diameter and recorded the species and MT (see Appendix S1: Table S1 for plot characteristics including dominant species). Four 0.1‐ha plots per MT (referred to as “EcM legacy” and “AM legacy” plots) were harvested in the winter of 2020–2021 and four were left as unharvested controls, for a total of eight 0.1‐ha plots in each control and treatment group (Appendix S1: Figure S1). To reduce potential differences among plot locations, we chose locations for control and treatment (harvest) plots with similar slopes, aspects, and dominant tree species within a MT. We measured plot characteristics before harvesting in 2020 across the 16 sites including soil moisture, pH, nutrient availability, and fungal biomass (described below), to employ the Before‐After Control‐Impact (BACI) framework with an equal number of paired control and treatment sites (Underwood, 1992).

To assess how harvesting affected soil conditions, we measured soil moisture, pH, fungal biomass, and nitrogen (N) availability before and after timber harvesting. We measured volumetric soil moisture with a probe (METER, Teros 10) at the three soil coring locations per plot in October 2020, June 2021, and June 2022. We chose these subplot locations to be approximately 5 m from the plot center using cardinal directions at least 90° apart to stratify sampling. To assess soil pH and mycorrhizal biomass, we collected 10 cm of the OA horizon prior to timber harvesting (October 2020) and following timber harvesting in both the early growing season (June 2021 and 2022) and at the end of the growing season (October 2021 and 2022) using a 5‐cm diameter soil core in triplicate at each plot after removing the leaf litter (Oi layer) if present. Soils were transported back to the lab on ice, where they were frozen at −20°C until processing. We analyzed soil percent carbon (C) and N on an elemental analyzer (EA‐IRMS; Thermo Scientific) to calculate the soil C:N ratio on preharvest soils. To measure soil pH, we used a 1:1 ratio of 5 g of MilliQ water and 5 g of thawed soil on a Mettler Toledo S220 pH/ion meter. Based on initial results showing a positive effect of mycorrhizal legacy for AM seedlings, we measured extractable calcium (Ca; Agricultural and Environmental Testing Lab, UVM Extension) on a composite of B horizon soils combined from three cores sampled in each plot in June 2024.

To measure the relative differences in nutrient availability, we measured available soil nitrogen (NH_4_ and NO_3_) and phosphate (PO_4_) using cation and anion exchange resins (Thermo Scientific), held in bags constructed using pantyhose (L'eggs Womens Everyday). We collected these data prior to timber harvesting (October 2020) and following timber harvesting in both the early growing season (June 2021 and 2022) and at the end of the growing season (October 2021). In each plot, we deployed triplicates of each resin bag type at the OA‐A horizon interface and at least 3 cm below the soil surface, for 1 month. Following field collection, we extracted resin bags using a 2 M KCl solution (Lajtha, 1988) and then measured extracts for respective nutrients on an auto analyzer (Lachat instruments, QuickChem 8500, Denver, CO, USA). For both soil sampling and resin deployment, we established permanent locations to resample near the same location when possible.

### Soil microbial community characterization

To assess harvest effects on soil microbial community composition, we combined an estimate of fungal biomass with internal transcribed spacer (ITS) sequencing to calculate biomass of fungal guilds within both the harvested and control plots. First, we measured the relative biomass of fungi by phospholipid fatty acid (PLFA) and neutral lipid fatty acid (NLFA) analyses, performed at the Soil Health Assessment Center at the University of Missouri (Buyer & Sasser, 2012). This method is applicable for quantifying the amount of fungal (Lewe et al., 2021) and AM fungal (NLFA; Lekberg et al., 2022) biomass. Measured NLFA values (16:1 w5c) represent AM fungal biomass (Kaiser et al., 2010; Olsson & Lekberg, 2022). Because sequencing data of communities are relative (Gloor et al., 2017), we used the fungal fatty acid signature 18:1 w9, which is widely used as a biomarker for non‐AM soil fungal biomass (Giray et al., 2024; Kaiser et al., 2010). Then to estimate EcM, saprotrophic, and pathotrophic fungal biomass, we multiplied these PLFA values by the summed relative abundances for each guild (based on sequencing described below) for each sample (Lewe et al., 2021). This is a key step in estimating EcM fungal biomass because EcM and saprotrophic fungi are not distinguishable by PLFA markers. As we used an AM NLFA biomarker to quantify AM biomass, we did not directly compare EcM and AM biomass with one another and instead analyzed them in separate models.

To characterize fungal community composition, we extracted DNA from OA horizon soils using the Qiagen PowerSoil Pro Kit (Qiagen Science Inc., Germantown, MD). DNA was sequenced at the Hubbard Center for Genome Studies, University of New Hampshire, for the fungal ITS2 region using ITS4‐FUN (AGCCTCCGCTTATTGATATGCTTAART) and 5.8S‐FUN (AACTTTYRRCAAYGGATCWCT) primers (Taylor et al., 2016). The first round of PCR amplified the ITS2 marker along with priming regions for the TruSeq read primers. We performed PCR in 12 μL reactions using 6 μL of a hot start, high‐fidelity polymerase (Kapa 2X HIFI HotStart ReadyMix), 0.7 μL of each primer (at 5 uM), and 2 μL of extracted DNA. Thermal cycling conditions included a 3‐min hot start at 94°C, 35 cycles of denaturing (94°C, 0:30), annealing (58°C, 0:30), and extension (72°C, 0:30), and a final extension of 7 min at 68°C. Successful amplification was verified using agarose gel electrophoresis (1.5%–2.0%). The first PCR reaction was cleaned with a 1:10 dilution. The second round of PCR added the P5 and P7 flow cell adapters to prepare the library for sequencing on an Illumina MiSeq, along with an external set of sample barcodes located between the flow cell adaptors and read primers. PCR was performed in 15‐μL reactions using 6 μL of a hot start, high‐fidelity polymerase (Kapa 2X HIFI HotStart ReadyMix), 5 μL of each primer (at 5 nM), and 2 μL of diluted product from the first PCR as template. Thermal cycling conditions were a 3‐min hot start at 94°C, 15 cycles of denaturing (94°C, 0:20), extension (72°C, 0:15), and a final extension of 7 min at 72°C. Amplicons were cleaned with the Qia‐quick PCR purification kit (Qiagen). Purified products were quantified using a Qubit 2.0 fluorometer with the Qubit dsDNA HS assay (Thermo Scientific, Grand Island, NY). Amplicons were pooled at equal concentration and sequenced on an Illumina MiSeq using V3 chemistry, using paired‐end sequencing (300 cycles). Sequences were separated by barcode at the Hubbard Center for Genome Studies, then filtered for quality and assigned to amplicon sequence variants (ASVs, equivalent to 100% identity operational taxonomic units) using the DADA2 program (Callahan et al., 2016) in R software. Non‐singleton ASVs were identified to the lowest confident taxonomic level using the naïve Bayesian classifier RDP using the UNITE database for ITS reads (Nilsson et al., 2019). Since exact sequence variants are not appropriate biological units for fungi (Tedersoo et al., 2022), we agglomerated ASVs to species level based on taxonomic identification.

While other regions such as LSU and SSU have been preferentially used over the ITS region for identifying AM fungi, some studies have shown that the ITS2 region has successfully characterized potential soil AM communities (Heeger et al., 2019; Kohout et al., 2014; Lekberg et al., 2018; Tedersoo et al., 2022). We matched ASVs with taxonomic and functional guild information using the FUNguild database (Nguyen et al., 2016) and assigned all fungal species to one of six broad putative categories: AM, EcM, plant pathogens/endophytes, saprotrophs, other, and unassigned. The “other” category consisted of species assigned to rarer categories (lichenized fungi, animal pathogens, etc.). We made no attempt to separate plant pathogens from other, nonpathogenic plant endophytes because even individual fungal strains can switch between these lifestyles based on plant host and environmental conditions. For species assigned to multiple categories, for analytical purposes we assigned them to a single guild with the following priority (EMF > plant pathogen/endophyte > saprotroph) for analytical purposes.

### Seedling survival and growth

From May 14 to 17, 2021, we planted 20 seedlings each of eight different species per plot, four AM species (*A. saccharum*, *Acer rubrum*, *Nyssa sylvatica*, and *Prunus serotina*), and four EcM species (*Quercus rubra*, *Carya cordiformis*, *Tilia americana*, and *Betula lenta*). We chose to include an equal number of AM and EcM‐associated species that aligned with species of interest from regional forest managers, as well as potential climate projections for the region. We split each plot down the slope and randomly assigned species planting locations across a 1 × 1 m grid, with one mycorrhizal association assigned to each half (Appendix S1: Figure S1). We allowed for 2 weeks of acclimatization before initial measurements to ensure seedlings did not die from planting shock. We then caged seedlings to reduce deer browse and measured volumetric soil moisture at each seedling location with a probe (METER, Teros 10).

We measured initial seedling height (to apical bud) and root collar diameter after planting and again at the end of each growing season, August 30–September 1, 2021 and August 29–August 31, 2022. We documented seedling survival (dead = 0, or alive = 1) and the “condition” of the terminal bud as intact or missing (1 = missing, 0 = intact). If a new leader had sprouted due to a missing terminal bud, we measured the height to the top of the new leader.

To estimate green biomass accumulation, we followed equations from Clark et al. (2022), where seedling volume was assumed to be conical and calculated based on the volume of a cone and calculated green biomass using constants from Miles and Smith (2009). Finally, we calculated the relative growth rates as the natural log of change in biomass from initial measurements (May 2021) to the end of the second season of growth (2022), divided by time from seedling planting to measurements at the end of the second season (Hunt & Cornelissen, 1997).

### Leaf ^15^N stable isotopes

To further investigate how access to available nitrogen could affect seedling survival and growth, we measured δ^15^N in seedling leaves after the first season of growth in fall 2021. The ratio of ^15^N/^14^N gives an indication of the nitrogen source, where a lower value of δ^15^N can be an indication of fractionation of N by mycorrhizal fungi as they transfer it to seedling roots (He et al., 2009; Hobbie & Högberg, 2012). We took duplicate hole punches from two seedlings per species within 8 m of the plot center and within 8 m of the plot edge in each plot. We foil‐balled duplicate ¼ cm diameter leaf punch samples and measured them on an isotope ratio mass spectrometer (IRMS, Thermo Scientific Flash EA with Delta V Advantage).

### Statistical analyses

We used R software (R Core Team, 2022) for all data formatting (packages included in “tidyverse,” Wickham et al., 2019) and analyses. We used linear mixed‐effects models (1) to investigate potential changes in AM, EcM, pathotrophic, and saprotrophic fungal biomass, soil nutrients, pH, and soil moisture from before and after timber harvesting and between control and harvested plots using the BACI design, (2) to test for the interactive effects of MT, or legacy, and seedling mycorrhizal association on seedling survival and growth, and (3) to test for differences in soil Ca between plot MTs following findings that AM seedlings had a survival benefit in the AM legacy plots.

Based on a priori hypotheses that nutrients, nitrogen (NH_4_ and NO_3_) and phosphate (PO_4_), and mycorrhizal biomass would vary between mycorrhizal legacies and over time following harvesting, we included a three‐way interaction between plot MT (AM vs. EcM), treatment (control vs. harvested), and sampling time (five time points including before harvest) using generalized linear mixed effects models (GLMMs). For all models, we included random intercepts for sampling location nested within the plot (16 plots, each with three sampling locations). We ran models for all responses except soil nutrients and pathotrophic biomass using the function “lme” from the package “nlme” (Pinheiro & Bates, 2000). We visually checked model assumptions and accordingly log‐transformed AM biomass to stabilize the residual variance. To account for zeros in the soil nutrient data potentially due to detection limits and pathotrophic biomass, we used the package and function “glmmTMB” with a ziGamma error distribution (Brooks et al., 2017). For each soil nutrient, we also included plot‐level soil moisture content as a covariate because variation in seasonal and yearly precipitation can affect pore water flow through the deployed resins. We visually checked nutrient model assumptions using randomized quantile residuals to check the GLMM assumptions (R package “DHARMa”; Dunn & Smyth, 1996; Hartig, 2016).

We tested for temporal autocorrelation in nutrient and fungal biomass models by adding a first order autoregressive correlation structure (function “CorAR1”) for errors over time, nested within sampling location. We then used Akaike information criterion (AIC) to compare models with and without a temporal correlation structure for the errors and determined that the autocorrelation component did not improve the fit. We also assessed spatial autocorrelation between sites by visualizing the relationship between site‐level model residuals and plot distance (i.e., latitude and longitude) with semivariograms (Appendix S1: Figure S2). To ensure that spatial autocorrelation did not affect our results, we compared model AIC for models without spatial autocorrelation and those with spatial autocorrelation. First, we added a spatial autocorrelation component using the exponential structure with glmmTMB. The mycorrhizal biomass models converged with this added structure, but had higher AIC (>2) values, and so we used the more parsimonious model versions without a spatial autocorrelation structure. Importantly, the results from both model versions had near identical statistical results. For all other data and models that did not converge with the above structure, we accounted for spatial autocorrelation by including plot nested within block, where we assigned plots within unit 14 to block “a,” plots within units 1 and 2 to block “b,” and the other AM plots on the east side of the forest as block “c” (see Appendix S1: Table S1 for “unit” and “plot” designations). There were no differences in model results, and adding the block increased AIC by two values (the cost of adding an additional term to the model), so we removed this component from final model versions (see Appendix S1: Table S2 for final models).

If there were significant interactions between time and harvest treatment or mycorrhizal legacy, we determined the changes between pre‐ and postharvest conditions by comparing each posttreatment year mean minus the pretreatment mean for both control and harvest treatments. This linear comparison removed the effect of time by subtracting the before‐after difference in the paired control plots and allowed an estimate of the treatment effect that does not include potential bias due to different environmental conditions across years. For example, a negative contrast value indicates a negative effect of the treatment after accounting for differences in environmental conditions between years using the control plots. All linear contrasts were done using the “emmeans” package in R (Lenth, 2021).

We analyzed seedling survival and growth for the 2021 and 2022 growing seasons. We used linear mixed models (LMMs, lme4 and lmerTest packages, function “lmer”) to analyze seedling growth and a generalized LMM (function “glmer”) with a binomial distribution (Bolker et al., 2009) to analyze seedling survival. For both models, we included plot and seedling species nested within mycorrhizal association as random effects and year as a fixed effect. Based on a priori hypotheses, we included a two‐way interaction between mycorrhizal legacy and seedling mycorrhizal association. To account for the spatial variability in soil moisture across each plot, we included percent soil moisture at the individual seedling level as a fixed effect. This soil moisture was averaged from three time points (June 2021, September 2021, and June 2022). Because damage to apical buds directly affects seedling growth and survival, we also included seedling condition (binary variable indicating an intact or broken apical bud) as a covariate. We also included initial height as a covariate. To analyze differences in seedling foliar ^15^N and percent N, we also used LMMs with a three‐way interaction between plot legacy type, seedling mycorrhizal association, and the seedling location in the plot (center vs. edge) with seedling species nested with mycorrhizal association and plot as random effects for each model. We assessed models for spatial autocorrelation as described above for BACI models (Appendix S1: Figure S2) and visually checked model assumptions.

To model soil Ca content, we used the function “lm” to test for the interaction between MT and harvest treatment only, as these soil samples were collected from one time point and from a composite sample per plot. We tested for spatial autocorrelation as described above for BACI models (Appendix S1: Figure S2) and visually checked model assumptions.

To characterize potential harvesting effects on fungal community composition between mycorrhizal legacies, we used the “vegan” package (Oksanen et al., 2020). Specifically, we created Bray–Curtis distance matrices of the fungal community abundances (function “vegdist”) and tested for an interaction between MT and harvest treatment using a permutational multivariate analysis of variance (PERMANOVA; function “adonis2”). We analyzed each year of data (2020, 2021, and 2022) separately due to separate sequencing runs. We visualized community differences using nonmetric multidimensional scaling (function “nmds”).

## RESULTS

Harvesting had fewer effects on soil chemistry than mycorrhizal legacy (see Table 1 for means and SEs and Appendix S1: Table S3 for full summary statistics). Although we expected soil N availability to increase after harvesting in treatment plots, both NO_3_ and NH_4_ decreased over time (GLMM, Est. = 4.5 mg g resin^−1^, *z* = 5.0, *p* <0.001; Est. = 0.9 mg g resin^−1^, *z* = 2.0, *p* = 0.050, respectively), similarly in control and treatment plots in the seasons following harvesting (Appendix S1: Table S3). PO_4_ availability also decreased following harvesting (GLMM, Est. = 1.4 mg g resin^−1^, *z* = 2.0, *p* = 0.047), in both control and treatment plots. Soil pH was higher on average in AM (5.57 ± 0.04) relative to EcM (4.98 ± 0.04) soils regardless of treatment or time following harvest (Appendix S1: Figure S3, MT, *F*_1,13_ = 10.4, *p* = 0.007). Soil moisture was higher in AM than EcM plots (LMM, *F*_1,19.4_ = 2.2, *p* = 0.037) and decreased across plots over time (LMM, *F*_1,131_ = 3.8, *p* < 0.001). Extractable Ca was higher on average in AM (868 ± 358 ppm) than EcM plots regardless of treatment (294 ± 76.1 ppm; *F*_1,12_ = 21.0, *p* = 0.004) and was similar between control and harvested plots (*F*_1,12_ = 0.1, *p* = 0.78).

**TABLE 1:** Soil variable means (±SE) for percent soil moisture (% moisture), phosphate (PO_4_), nitrate (NO_3_), ammonium (NH_4_), and pH for each mycorrhizal type, treatment, and sampling date (*n* = 48 per sampling date).

Mycorrhizal legacy	Treatment	Sampling date	Moisture (%)	NO_3_ (mg g resin^−1^)	NH_4_ (mg g resin^−1^)	PO_4_ (mg g resin^−1^)	pH
AM	Control	Fall 2020	30.8 (1.5)	1.0 (0.22)	0.17 (0.02)	0.09 (0.04)	5.5 (0.1)
		Summer 2021	30.9 (2.4)	0.03 (0.01)	0.25 (0.06)	0.07 (0.02)	NA
		Fall 2021	30.6 (2.1)	0.04 (0.02)	0.20 (0.05)	0.08 (0.06)	NA
		Summer 2022	35.2 (2.0)	0.01 (0.00)	0.00 (0.00)	0.00 (0.00)	5.6 (0.1)
		Fall 2022	32.4 (1.3)	NA	NA	NA	5.5 (0.1)
	Logged	Fall 2020	34.6 (2.5)	0.61 (0.04)	0.15 (0.01)	0.19 (0.10)	5.7 (0.2)
		Summer 2021	35.8 (3.8)	0.05 (0.01)	0.16 (0.03)	0.09 (0.02)	NA
		Fall 2021	31.3 (3.3)	0.05 (0.02)	0.14 (0.02)	0.07 (0.04)	NA
		Summer 2022	31.4 (1.8)	0.01 (0.00)	0.00 (0.00)	0.00 (0.00)	5.6 (0.1)
		Fall 2022	33.0 (2.6)	NA	NA	NA	5.6 (0.1)
EcM	Control	Fall 2020	40.3 (3.6)	0.45 (0.02)	0.17 (0.02)	1.39 (0.60)	4.7 (0.2)
		Summer 2021	30.0 (4.1)	0.03 (0.01)	0.31 (0.06)	0.06 (0.02)	NA
		Fall 2021	26.7 (4.7)	0.03 (0.01)	0.24 (0.04)	0.00 (0.00)	NA
		Summer 2022	37.2 (3.9)	0.00 (0.00)	0.00 (0.00)	0.00 (0.00)	4.8 (0.1)
		Fall 2022	33.1 (4.4)	NA	NA	NA	4.9 (0.1)
	Logged	Fall 2020	36.3 (2.8)	0.48 (0.010)	0.20 (0.050)	0.18 (0.08)	5.1 (0.2)
	Logged	Summer 2021	33.5 (2.5)	0.08 (0.04)	0.33 (0.07)	0.14 (0.07)	NA
	Logged	Fall 2021	36.5 (7.0)	0.09 (0.06)	0.30 (0.05)	0.00 (0.00)	NA
	Logged	Summer 2022	38.8 (1.6)	0.01 (0.00)	0.00 (0.00)	0.00 (0.00)	5.2 (0.2)
	Logged	Fall 2022	32.3 (1.3)	NA	NA	NA	5.1 (0.1)

*Note*: Fall 2020 sampling was prior to timber harvesting.

Abbreviations: AM, arbuscular mycorrhizal; EcM, ectomycorrhizal.

**FIGURE 2:**
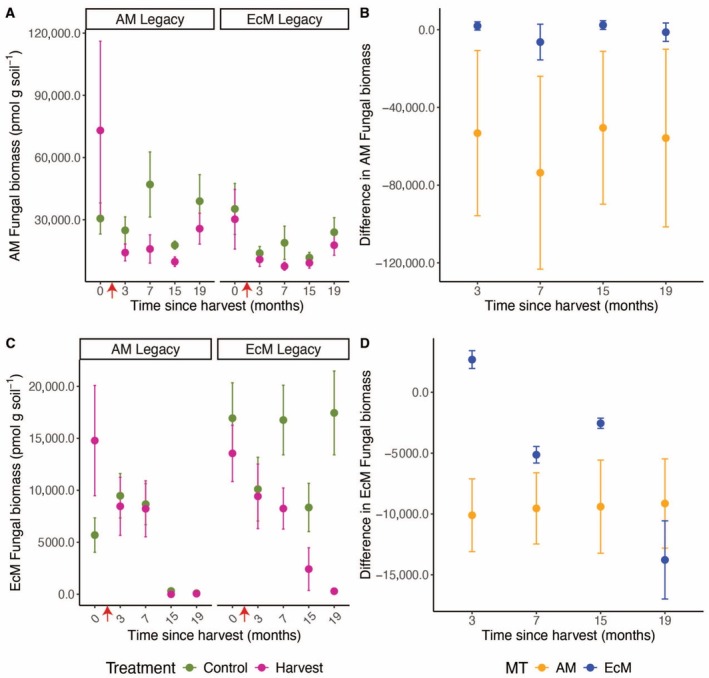
Mycorrhizal biomass changes over time before and after harvesting. Arbuscular mycorrhizal (AM) fungal biomass in panel (A) and ectomycorrhizal (EcM) biomass in panel (C), where mean values at each sampling time point for control and treatment plots and the harvest event is denoted by the red arrow on the *x*‐axes. AM biomass in panel (B) and EcM biomass in panel (D) show differences between mean control and treatment values for mycorrhizal legacy types (MT) across each time point. Error bars show SEs.

Timber harvesting affected fungal communities, but generally these effects were similar in both mycorrhizal plot types. Specifically, timber harvesting reduced AM fungal biomass relative to control plots in both mycorrhizal legacy plot types for the first 15 months (LMM, treatment [Trt] × time; Figure 2A,B; Appendix S1: Tables S4 and S5 for pairwise contrasts). Timber harvesting consistently reduced EcM fungal biomass similarly between AM and EcM legacy plots (LMM Trt × time; Figure 2C,D; Appendix S1: Table S4), with the greatest reduction in the fall of 2023 (Appendix S1: Table S5). Harvesting did not affect saprotrophic fungal biomass in either AM or EcM legacy plots or their interaction with time, but generally saprotrophic biomass was lower over time (LMM; Appendix S1: Figure S4 and Table S4). Pathotrophic biomass was moderately significantly (*p* < 0.1) higher in AM plots than EcM plots in both harvested and control plots (GLMM; Figure S4 and Table S4). AM and EcM plots harbored different soil fungal communities prior to harvesting in 2020, and the effect of mycorrhizal plot type continued to dominate fungal community composition in both harvested and control plots across sampling time points (PERMANOVA, MT × Trt; Appendix S1: Table S6 and Figure S5).

**FIGURE 3:**
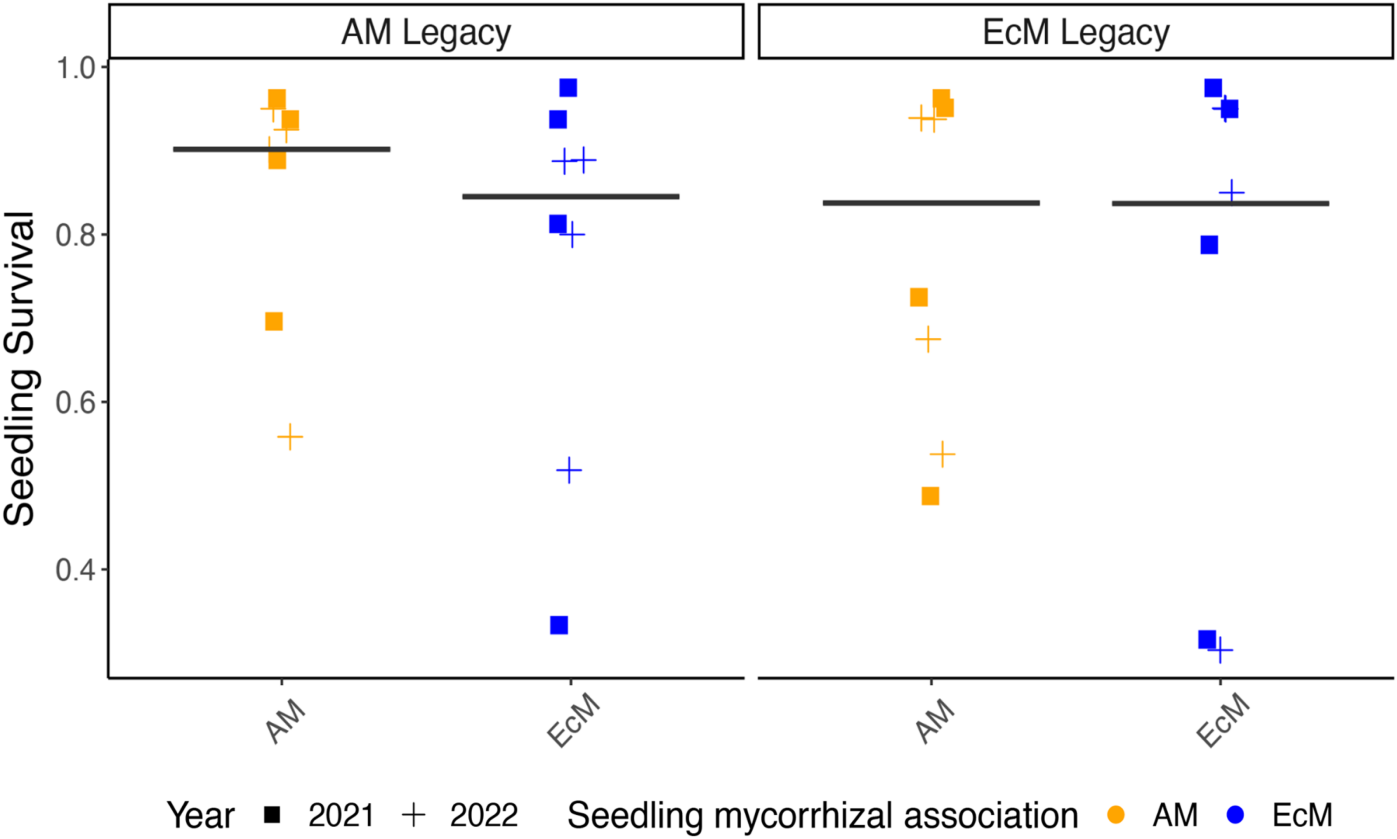
Seedling survival from planting in May 2021 to end of August 2022 in harvested 0.1 ha gaps that were previously dominated by arbuscular mycorrhizal (AM)‐associated (“AM Legacy”) or ectomycorrhizal (EcM)‐associated (“EcM Legacy”) trees. Points are jittered for visibility and represent mycorrhizal legacy‐seedling mycorrhizal association means for each year for percent survival (*n* = 80 per survival mean). Horizontal bars show the model prediction for average survival, where other model covariates are held at their mean value.

**FIGURE 4:**
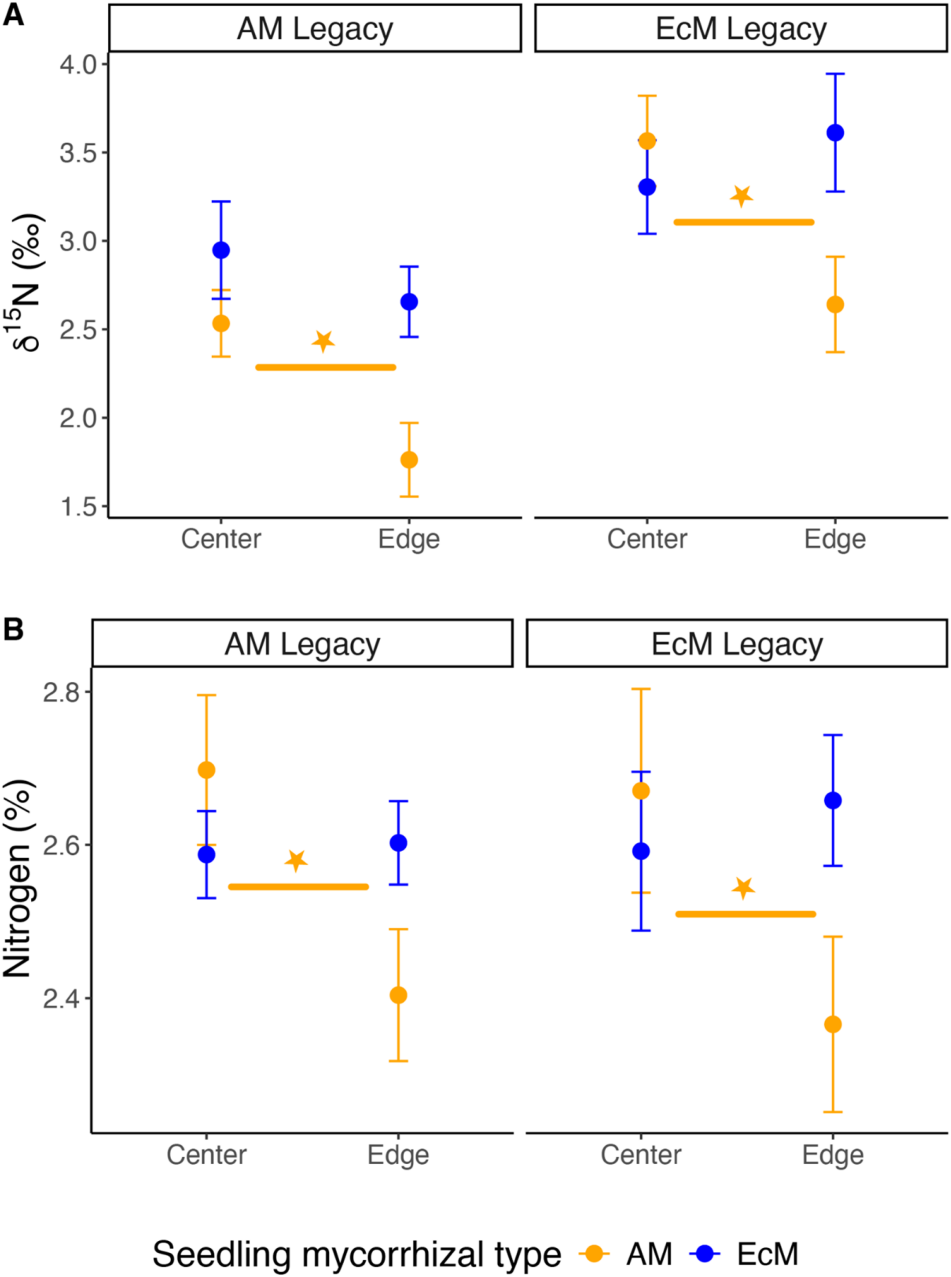
Foliar δ^15^N (‰) (A) and percent N (B) differed for arbuscular mycorrhizal (AM)‐associated seedlings between center and edge locations. Points show average values across seedlings from each associated mycorrhizal type, either AM or ectomycorrhizal (EcM), position in harvested gap (either within an 8 m radius of center or within 8 m of the plot edge), and plot mycorrhizal legacy (*n* = 51 per group × 8 groups for a total of 408 samples). Error bars represent SE, colors designate seedling mycorrhizal type, and horizontal lines with stars show significant (*p* < 0.05) differences between groups.

The mycorrhizal legacy was important for AM, but not EcM seedling survival. After two growing seasons, AM seedlings had 51% (mean ± 21%) greater survival when planted in AM compared to EcM legacy plots (LMM, MT × MST, *F* = 5.2, *p* = 0.022; Figure 3). However, there were no legacy effects on seedling growth (LMM, MT × MST, *F* = 1.1, *p* = 0.571; Appendix S1: Table S7). Mycorrhizal legacy also affected foliar N in AM, but not EcM seedlings. After one season of growth, foliar δ^15^N (‰) was depleted (LMM, Position × MST, *F*_390.8_ = 9.5, *p* = 0.002) and foliar percent N was greater (LMM, Position × MST, *F*_1,390.8_ = 14.6, *p* = 0.001; Figure 4) in AM relative to EcM seedlings planted in the outer 8 m in both AM and EcM plots.

## DISCUSSION

We present one of the first studies to show how timber harvesting affects both EcM and AM fungal biomass in temperate forests. Specifically, in legacy plots for both mycorrhizal types, AM fungal biomass experienced a significant decrease after the first season following timber harvesting, while EcM fungal biomass continued to decline to nearly a 100% decrease over 15 months. Harvesting likely decreased EcM fungal biomass to this degree because the C supply from their symbionts was cut off (Borgmann‐Winter et al., 2022; Castaño et al., 2020; Högberg & Högberg, 2002). We also found that harvesting affected fungal community composition, in line with other studies showing effects of timber harvesting on EcM fungal and free‐living communities (Jones et al., 2003; Kohout et al., 2018).

While harvesting initially affected the biomass of AM fungi more than EcM fungi, AM fungal biomass began to recover after 2 years, suggesting AM fungi may have mechanisms that promote a faster recovery than EcM fungi in managed landscapes. Previous work on mycorrhizal spore dispersal into harvested gaps by rodents showed that AM fungal spores were dispersed by a wide variety of rodent species relative to a single rodent species that dispersed EcM fungal spores (Stephens et al., 2021; Stephens & Rowe, 2020). Furthermore, early colonizing herbaceous and shrub species such as *Rubus* sp. are AM‐associating and could have supported AM fungi establishment in conjunction with spore dispersal (Grünfeld et al., 2020). These ruderal AM plant species could also support early AM colonization and establishment from wind‐dispersed fungal spores (Borgmann‐Winter et al., 2023; Chaudhary et al., 2020).

Contrary to our predictions that EcM‐associated seedlings would benefit from being planted in an EcM legacy plot, we found no legacy effect on EcM seedling success. This result is surprising as some studies show that EcM seedlings benefit from potential access to mycorrhizal hyphae when planted in soil near con‐mycorrhizal adults (Liang et al., 2020, 2021). However, species‐specific responses may be important given other research where increased proximity to adults of the same mycorrhizal association had a neutral to negative effect on EcM seedling success (Jevon et al., 2020, 2022; Kadowaki et al., 2018). One possible explanation for this result is that the hyphae of many EcM fungal species can grow relatively greater distances than AM fungi (See et al., 2019). However, given our results showing a 90% decrease in EcM fungal biomass, and other research suggesting that EcM fungal mycelium is restricted to patches less than 3 m in diameter (Cortese & Horton, 2024), it is unlikely that EcM fungal hyphae could have reached across our 0.1‐ha gaps. Our results suggest that EcM seedlings depend less on access to mycorrhizal symbionts in their early growth stages in Northeastern forests or that the large decline in EcM fungal biomass after harvesting negated any mycorrhizal‐derived benefit of being planted in an EcM legacy plot.

The benefits of growing in AM legacy plots outweighed the potential negative effects of fungal pathogen exposure on AM seedlings even though the AM legacy plots had five times the estimated fungal pathotroph biomass as the EcM legacy plots. This effect is in opposition to previous studies showing that pathogen load near adult conspecifics negatively affects AM seedlings more than EcM seedlings (Liang et al., 2020, 2021). Despite AM‐associated species such as *P. serotina* experiencing higher mortality with increasing native pathogen load (Liang et al., 2016; Reinhart et al., 2003), in our study, *P. serotina* grew better in AM legacy plots. One explanation for this AM legacy effect is that AM fungi may have mitigated this potential increased pathogen exposure (Liang et al., 2015) through biocontrol mechanisms that reduced pathogen load on or near AM roots (Azcón‐Aguilar & Barea, 1997). The benefits of AM fungal nutrient provisioning (Koide, 1991) could have also outweighed this pathogen exposure.

Our results showing depleted foliar δ^15^N in AM seedlings planted near gap edges, but not EcM seedlings, indicate that AM seedlings at the edges of gaps may have had better access to fungal inoculum and thus received more N via mycorrhizal fungi. Both EcM and AM fungi have been shown to fractionate N, retaining the heavier ^15^N and passing the lighter ^14^N to tree hosts (Etcheverría et al., 2009; Hobbie & Högberg, 2012), but this fractionation has been found to be stronger for EcM‐ (−2.3‰ ± 0.2‰) compared to AM‐associated plants (−1.1‰ ± 0.1‰) (Craine et al., 2009). Because AM fungi preferentially take up nitrate (Ngwene et al., 2013), which becomes depleted in ^15^N relative to ammonia during nitrification (Gurmesa et al., 2022), greater nitrification following timber harvesting (Pardo et al., 2002) could also contribute to this difference in foliar ^15^N. However, N mineralization rates were likely higher in the center relative to plot edges due to greater fluctuations in microclimate and heightened litter decomposition (Jerabkova et al., 2011; Thiel & Perakis, 2009). In that case, there should have been more ^15^N‐depleted nitrate available to AM seedlings in the plot center, which is the opposite pattern of our results. Despite this δ^15^N pattern suggesting AM fungal fractionation, AM seedlings at gap edges also had lower foliar percent N than seedlings at gap centers. These lower N values highlight that competition for soil N with nearby adult trees may have outweighed the potential benefits of N transferred from mycorrhizal fungi at the plot edges (Legge et al., 2025; Teste & Simard, 2008).

An alternative mechanism to mycorrhizal nutrient facilitation is that the positive effect of an AM legacy on AM seedlings could be driven by preexisting abiotic conditions, including soil nutrient availability and chemistry. AM tree traits can be more sensitive to certain soil characteristics than EcM species (Midgley et al., 2015; Zhang et al., 2018). Surprisingly, we found no detectable difference in soil N or phosphorus availability following harvesting or between MTs, suggesting that other soil conditions contributed to AM seedlings benefitting from an AM legacy. In contrast, higher soil pH in AM relative to EcM soils, regardless of harvest treatment, supports the hypothesis that preexisting soil chemistry differences between AM and EcM mycorrhizal legacies can contribute, or coincide with, other edaphic characteristics that contribute to seedling success (Carrino‐Kyker et al., 2016; O'Brien et al., 2011).

Greater soil Ca in AM soils may have facilitated AM tree dominance prior to harvesting (Wagenknecht et al., 2023) and could in part be driving AM seedling success (see Appendix S1: Figure S6 for a visual relationship between plot Ca and average seedling survival). Soil Ca is an essential nutrient for arbuscule formation in AM seedling roots (Weber & Claus, 2000) and has been documented as a driver of growth and survival for two of the dominant AM tree genera at our study site, *Acer* and *Fraxinus* (Momen et al., 2015; Weber & Claus, 2000). In Northeastern forests, Ca loss has been identified as a primary driver of sugar maple decline (Cleavitt et al., 2018). When forests experiencing soil Ca depletion were fertilized with CaCO_3_, trees, particularly the AM tree *A. saccharum*, had marked increases in growth (Battles et al., 2014; Juice et al., 2006; Long et al., 2022). Underlying bedrock and weathering processes predominantly control Ca availability (Naples & Fisk, 2010). Trees can reinforce these differences by transporting Ca from deeper soil zones to surface soils via roots (Dijkstra & Smits, 2002) and by controlling Ca losses through their litter chemistry effects on pH (Lin et al., 2022). We hypothesize that greater soil Ca availability in AM relative to EcM legacy soils could have contributed to the AM seedling homefield advantage, and suggest that more work is needed to disentangle the effects of biotic and abiotic drivers associated with mycorrhizal legacies.

Our findings show strong effects of harvesting on mycorrhizal fungal biomass, but not soil available nitrogen, and have implications for reforestation strategies in temperate forests. We suggest that AM fungal biomass can begin to recover after two growing seasons following gap harvesting up to 0.1 ha. Although we are unable to provide a prediction for EcM fungal biomass recovery given the length of this study, other research has shown that retention of some mature canopy trees in harvested areas can maintain nearly all EcM fungal species present in an intact forest (Parladé et al., 2019; Tomao et al., 2020). Our mycorrhizal legacy results suggest that EcM‐associated seedlings are likely not limited by the distance from adult EcM trees at the scale of 0.1 ha, as suggested in other studies (Simard et al., 1997, 2021), and could be planted in soils regardless of the mycorrhizal legacy in gaps of similar size in temperate forests. Second, planting AM‐associated seedlings in soils previously dominated by AM‐associated adult trees could increase seedling success; however, AM seedlings located closer to gap edges may experience greater competition for resources as demonstrated by their lower foliar N content. Given this potential edge effect, future research could examine how harvest gap size affects seedling success between mycorrhizal associations. The soil mineral nutrient content is also likely to be an important consideration for planting. Nutrient availability at a given site could create conditions where competition or mycorrhizal facilitation play a greater role in how seedlings fare (Van Nuland et al., 2023).

Forest communities will continue to shift due to altered rates of precipitation, changes in temperature, N deposition (Cleavitt et al., 2018; Jo et al., 2019), or from invasive insect outbreaks such as the emerald ash borer across the northern and eastern United States (Morin et al., 2017). Because these changes also coincide with shifts in either AM or EcM tree dominance, it is necessary to understand how mycorrhizal legacies affect seedling success (Policelli et al., 2020). Our findings that an AM legacy enhanced AM seedling success suggest that the expansion of AM‐associated trees across the eastern United States (Jo et al., 2019) will perpetuate further AM dominance through a positive feedback loop—via mycorrhizal facilitation, soil nutrient conditions, or likely the combination of both these biotic and abiotic drivers (Van Nuland et al., 2023).

## AUTHOR CONTRIBUTIONS

Amelia Fitch prepared the original draft, contributed to funding acquisition, conceptualization of study design, methodology development, data collection, data analysis, and visualization. Sarah Goldsmith contributed to conceptualization of study design, methodology development, data collection, and reviewing and editing. Eva O.L. Legge contributed to conceptualization of study design, data collection, and reviewing and editing. Audrey Adamchak contributed to data collection and reviewing and editing. Dustin Gannon contributed to methodology development and reviewing and editing. Alexandra M. Kosiba and Kevin Evans contributed to funding acquisition and reviewing and editing. Anthony W. D'Amato contributed to funding acquisition, conceptualization of study design, and reviewing and editing. Caitlin Hicks Pries contributed to funding acquisition, conceptualization of study design, methodology development, data collection, data analysis and visualization, and reviewing and editing.

## CONFLICT OF INTEREST STATEMENT

The authors declare no conflicts of interest.

## Supporting information


Appendix S1.

## Data Availability

Data (Fitch et al., 2026) are available in Dryad at https://doi.org/10.5061/dryad.18931zd75.
