# Supplementary material for: Mycorrhizal legacy mediates seedling success following timber harvesting in Northeastern forests
Source: Ecol Appl. 2026 Jul 8;36(5):e70281. doi: 10.1002/eap.70281 (PMC13345700; doi:10.1002/eap.70281)
Supplement: Supplementary file 1 — Appendix S1. [file EAP-36-e70281-s001.pdf]

Appendix S1

Mycorrhizal legacy mediates seedling success following timber harvesting in Northeastern forests

Amelia Fitch, Sarah Goldsmith, Anthony W. D'Amato, Eva O.L. Legge, Audrey Adamchak, Dustin Gannon, Alexandra M. Kosiba, Kevin Evans, Caitlin Hicks Pries

Ecological Applications

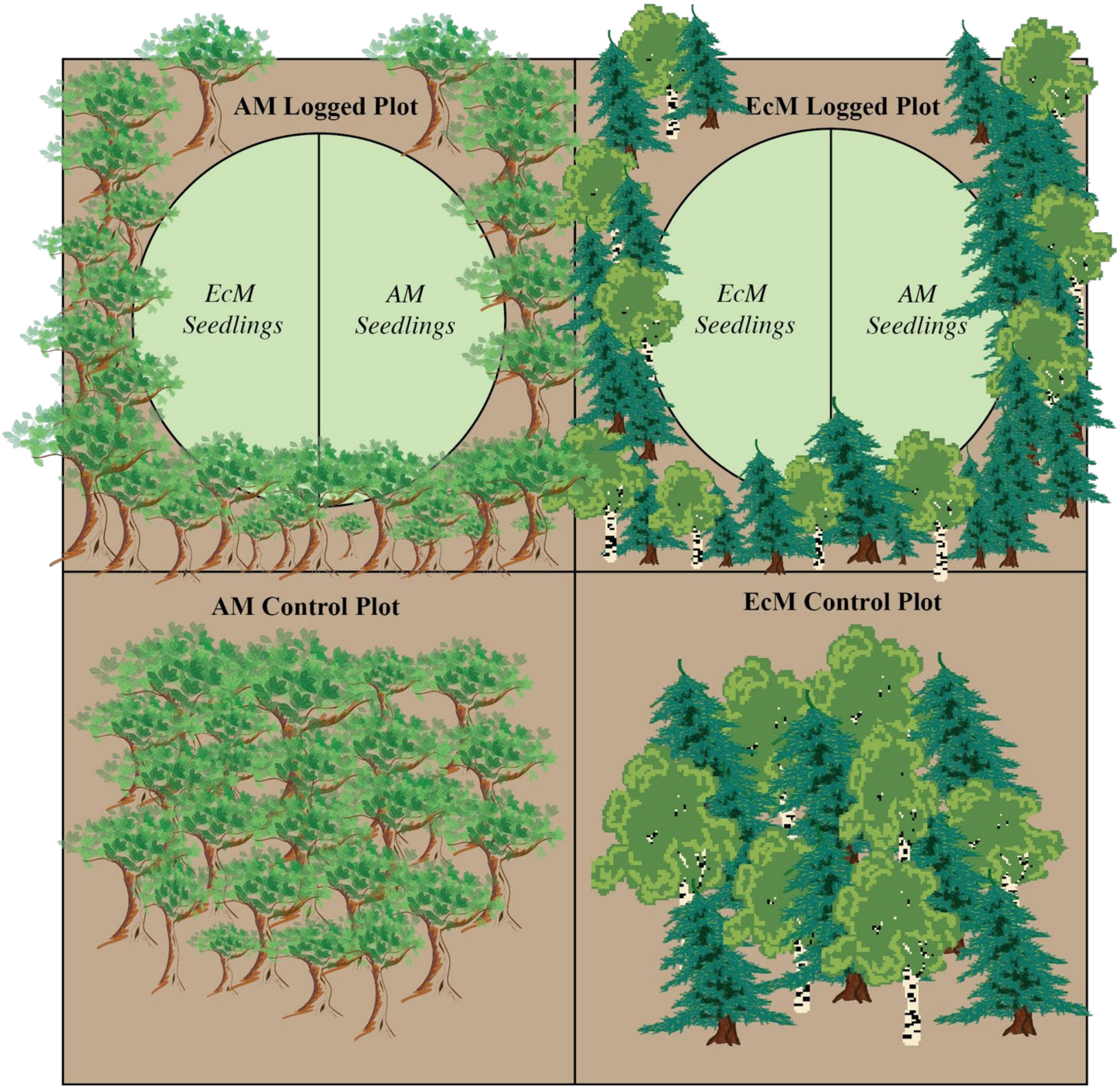

Figure S1. Eight 0.1 ha (18 m radius) gap-cut plots were established in the winter of 2021, four as AM-legacy and four as EcM legacy plots. These clear cuts were planted with AM and EcM-associated seedlings in May-June 2021, after which their survival and growth was routinely monitored. Eight 0.1 ha control plots were also established: four in EcM-dominated and four in AM-dominated stands. Figure created by Amelia Fitch and Eva Legge. This figure was reproduced from Fitch et al. (2022).

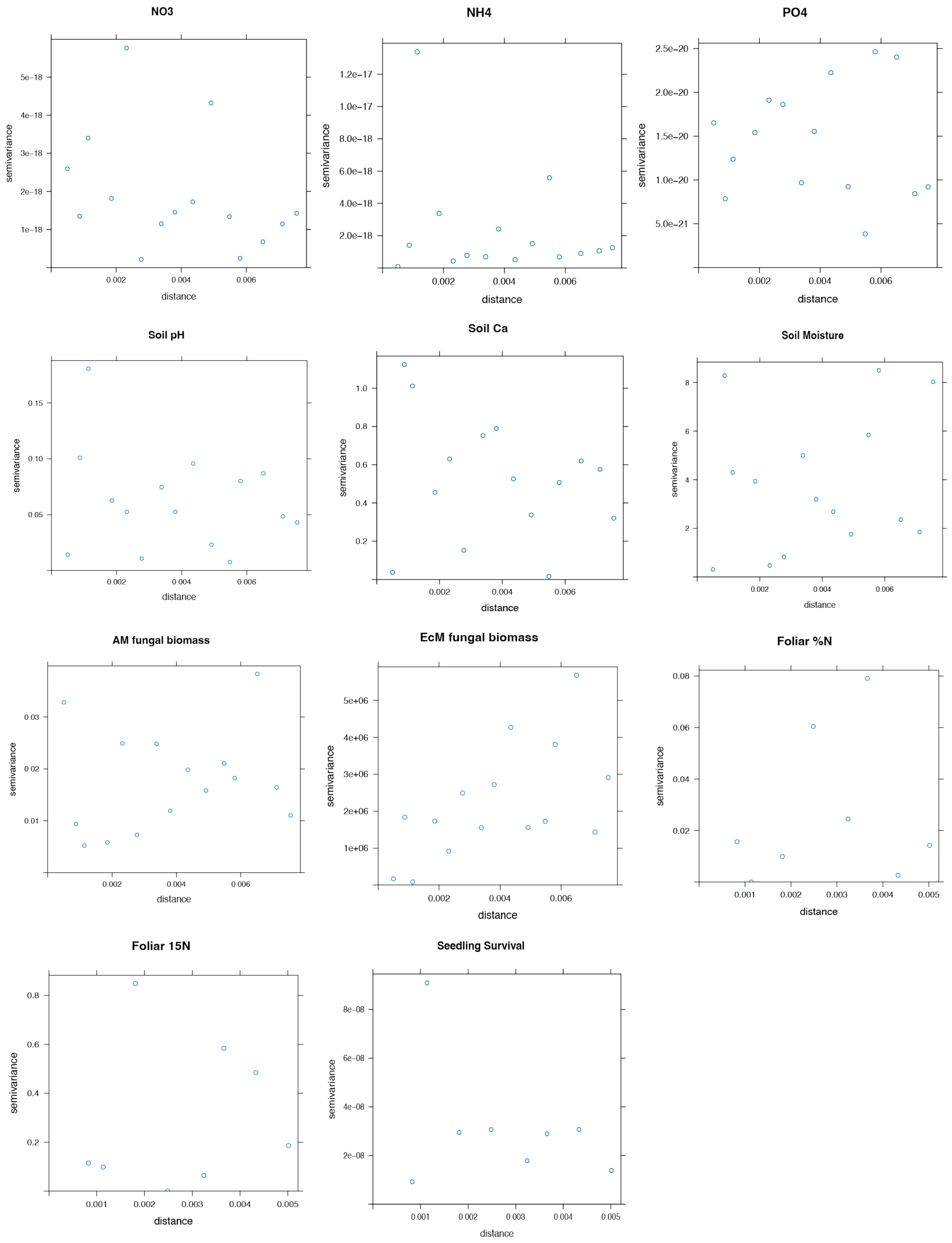

Figure S2. Semivariograms showing plot-level model residuals on the y axis and distance (i.e. latitude and longitude) on the x axis for soil chemistry, fungal biomass, and seedling survival.

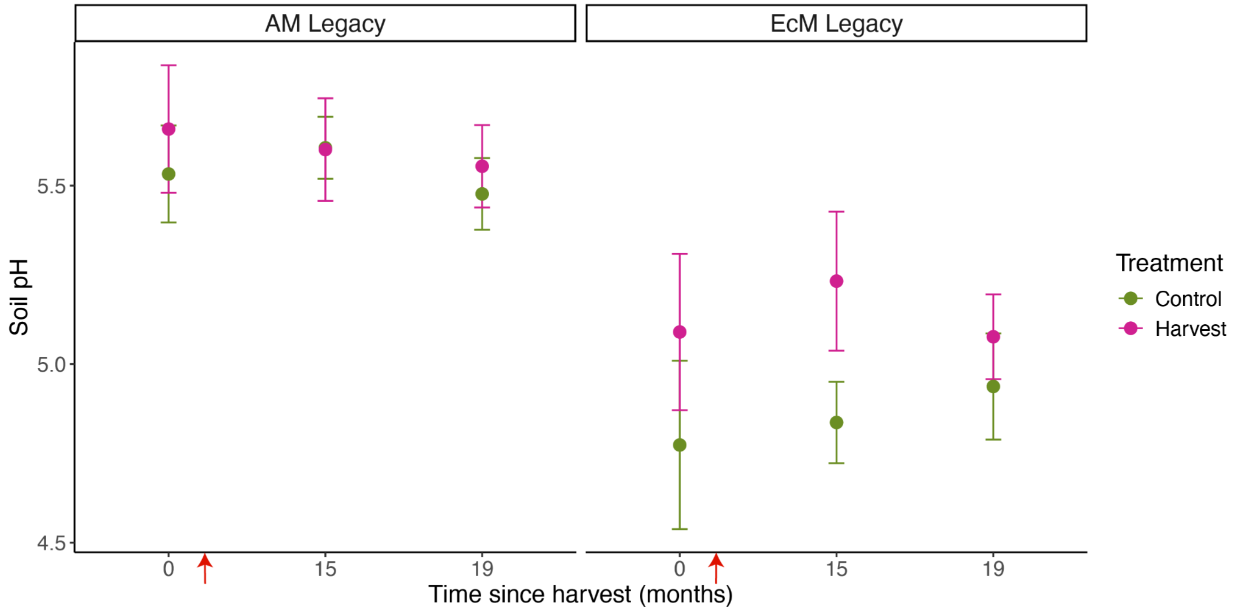

Figure S3. Soil pH, where points are means representing each mycorrhizal legacy, treatment, and at each sampling point, excluding 2021. Error bars show standard error.

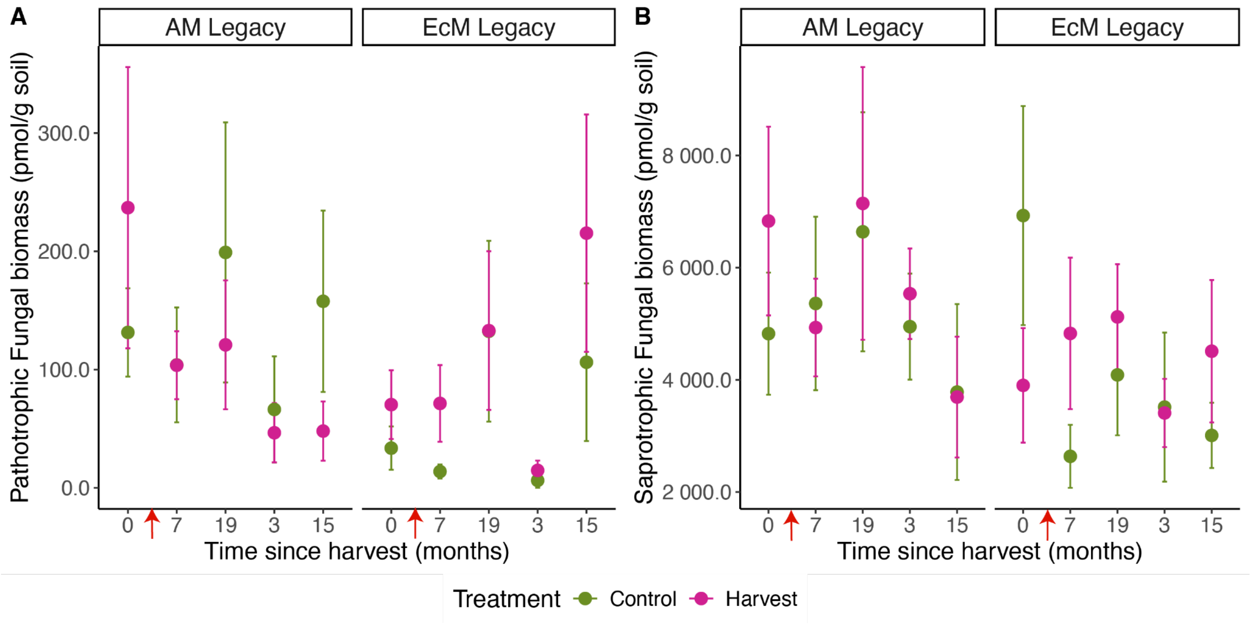

Figure S4. Pathotrophic (A) and saprotrophic (B) fungal biomass. The harvest event is denoted by the red arrow on the x-axes. Points show mean values at each sampling time point for control and treatment plots, the harvest event is denoted by the red arrow on the x-axes, and error bars show standard error. Fungal biomass values for respective functional groups were calculated by multiplying fungal phospholipid fatty acid values by the relative abundance of assigned ASVs.

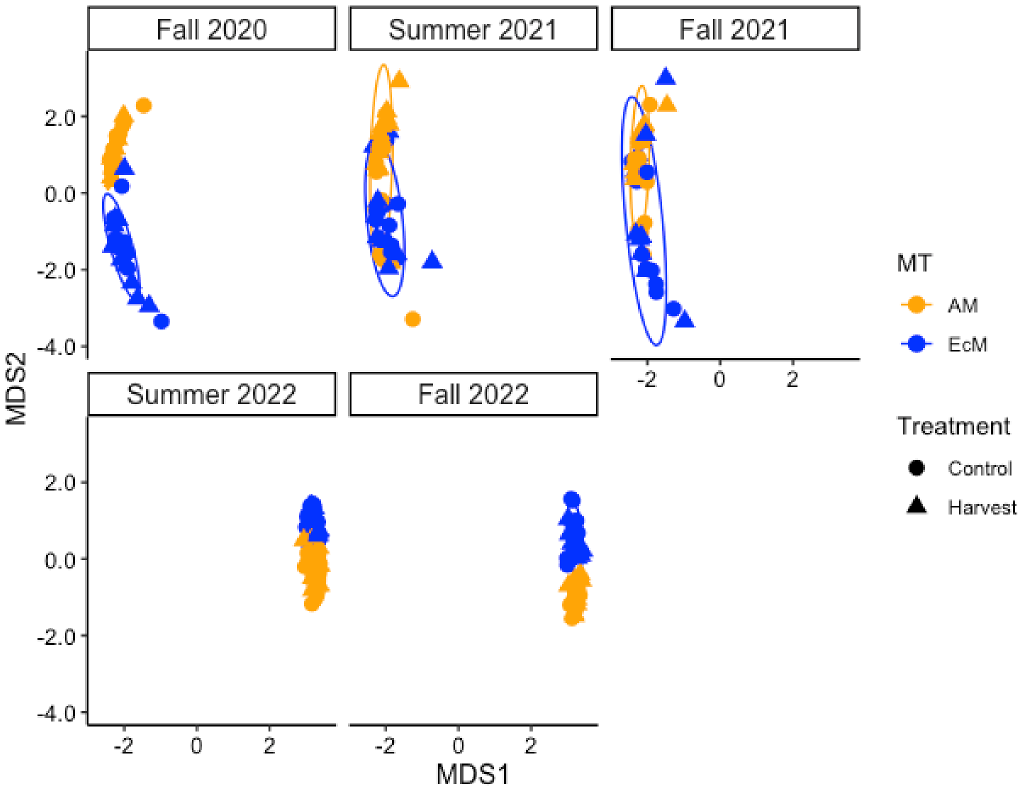

Figure S5. Non-metric multidimensional scaling (NMDS) representation of the absolute abundances of all fungal taxa, where points are faceted by time. Absolute abundances were calculated by multiplying fungal phospholipid fatty acid values by relative abundance values for all taxa except arbuscular mycorrhizal fungi, which were adjusted with neutral lipid fatty acid values. Points represent individual soil samples, where most of the 16 total plots contained three replicate samples, except where data were removed prior to rarefaction due to low numbers of sequences. Ellipses represent 95% confidence intervals.

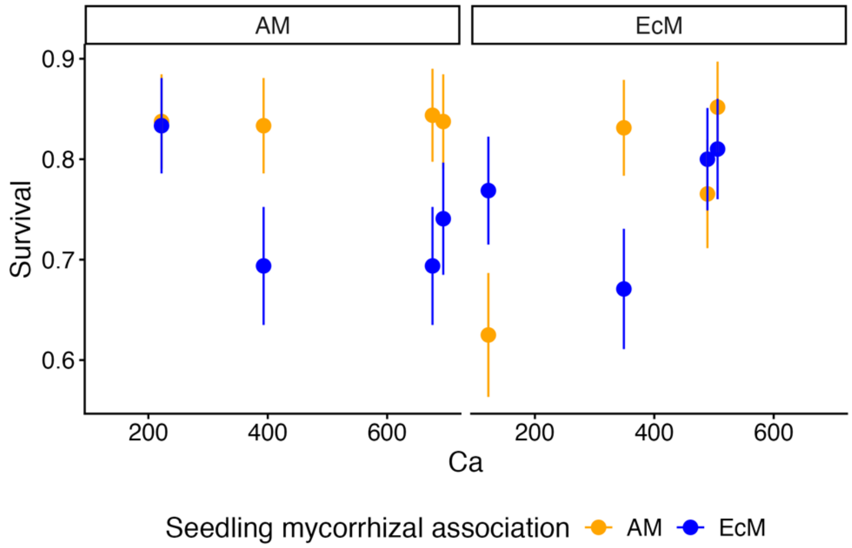

Figure S6. Soil calcium values for each plot and average seedling survival between mycorrhizal legacy plots. Colors show seedling mycorrhizal associations. Error bars show standard error around averaged seedling survival.

Table S1. Plot characteristics, where the plot ID contains both unit and plot number. MT is the plot dominant mycorrhizal type, and the dominant tree species is the highest basal area (BA) represented by one tree species, with the percent BA in parentheses. Treatment indicates whether the plot was gap harvested (quarter acre) or a control. Soil C:N and bulk density show mean ± standard error from three plot replicates prior to harvesting in fall 2020.

Plot	Lat/Long	MT	Dominant species	Treatment	Aspect	Soil C:N	Bulk density
1_1	-72.3232, 44.0504	EcM	Fagus grandifolia (44.5)	Control	S	24.23 (0.75)	0.19 (0.06)
1_10	-72.3252, 44.0494	EcM	Ostrya virginiana (45.4)	Control	SE	21.34 (0.36)	0.28 (0.03)
10_5	-72.3149, 44.0502	AM	Acer saccharum (62.2)	Harvest	SE	13.98 (0.69)	0.41 (0.02)
12_11	-72.3129, 44.0493	AM	Acer saccharum (54.2)	Control	SE	13.41 (0.27)	0.33 (0.04)
13_5	-72.3289, 44.0432	AM	Acer saccharum (84.5)	Control	NW	14.24 (0.44)	0.44 (0.01)
14_1	-72.3194, 44.0525	EcM	Tsuga canadensis (66.2)	Control	W	26.0 (0.89)	0.31 (0.04)
14_2	-72.3195, 44.0489	EcM	Tsuga canadensis (57.1)	Control	W	22.64 (1.59)	0.19 (0.02)
14_5	-72.3197, 44.0516	EcM	Tsuga canadensis (53.9)	Harvest	W	28.78 (0.43)	0.29 (0.05)
14_6	-72.3199, 44.0508	EcM	Tsuga canadensis (62.2)	Harvest	W	24.21 (3.41)	0.4 (0.14)
14_7	-71.3194, 44.0498	EcM	Tsuga canadensis (54.2)	Harvest	W	20.78 (0.21)	0.18 (0.02)

2_5	-72.3271, 44.0476	EcM	Fagus grandifolia (73.1)	Harvest	E	20.62 (0.66)	0.34 (0.06)
2_9	-72.3279, 44.046	AM	Acer saccharum (53.7)	Harvest	E	15.32 (0.46)	0.23 (0.06)
3_5	-72.3165, 44.0521	AM	Acer saccharum (67.7)	Harvest	SW	13.19 (0.95)	0.18 (0.04)
4_10	-72.3162, 44.0517	AM	Acer saccharum (66.2)	Control	SW	12.86 (1.24)	0.4 (0.01)
6_5	-72.3105, 44.0573	AM	Fraxinus americana (57.1)	Control	S	11.72 (0.40)	0.33 (0.04)
8_1	-72.3135, 44.055	AM	Fraxinus americana (53.9)	Harvest	SE	12.86 (0.72)	0.47 (0.03)

Table S2. Models for fungal biomass including AM, EcM, saprotroph and pathogens, soil nutrients, pH, and seedling growth and survival. Shorthand for model predictors are mycorrhizal plot legacy type (MT, AM vs EcM), mycorrhizal seedling type (MST, AM vs EcM), treatment (Trt, control vs harvested). The time predictor is a factor with five levels including pre-harvesting sampling. For fungal biomass, nutrients, and pH, sampling location (3 levels) was nested within plot (16 levels), for a total of 48 data points. Soil moisture in the soil nutrient model represents the three plot sampling locations co-located with resin bags, while soil moisture in the seedling survival and growth models are at the seedling level. Seedling condition is a binary variable representing whether seedlings had damage to the apical bud.

**Response variable**	**Model predictors**	**Model type**	**Random effect structure**
AM biomass	MT * Trt* Time	LMM	(1 | Plot) + (1 | Plot : Location)
EcM biomass	MT * Tr t* Time	LMM	(1 | Plot) + (1 | Plot : Location)
Saprotroph bimoass	MT * Trt * Time	LMM	(1 | Plot) + (1 | Plot : Location)
Pathotroph biomass	MT * Trt * Time	GlmmTMB	(1 | Plot) + (1 | Plot : Location)
Soil nutrients	MT * Trt * Time + soil moisture	GlmmTMB	(1 | Plot) + (1 | Plot : Location)
Soil pH	MT * Trt * Time	LMM	(1 | Plot) + (1 | Plot : Location)
Soil calcium	MT * Trt	LM	NA
Seedling survival	MT * MST + year + soil moisture + condition	GLMM	(1 | Plot) + (MST | Seedling species)
Seedling growth	MT * MST + year + soil moisture + condition	LMM	(1 | Plot) + (MST | Seedling species)

Table S3. Summary statistics for nitrate, ammonium, phosphate, and soil moisture. Time represents months post-harvest, MT represents mycorrhizal plot type, and treatment represents either control or logged plots.

NO_3 (mg/g resin)	z value	Pr(>|z|)
MT	1.0	0.307
Treatment	1.2	0.207
Time	5.0	< 0.001
% moisture	0.4	0.722
MT : Treatment	1.0	0.67
MT : Time	-0.6	0.583
Treatment : Time	-0.2	0.79
MT : Treatment : Time	-0.5	0.641
**NH_4 (mg/g resin)**	**z value**	**Pr(>|z|)**
MT	-0.8	0.395
Treatment	0.6	0.533
Time	2.0	0.050
% moisture	-1.0	0.328
MT : Treatment	-0.5	0.60
MT : Time	-0.7	0.491
Treatment : Time	0.4	0.72
MT : Treatment : Time	-0.4	0.697

Table S3 (continued)

**PO_4 (mg/g resin)**	**z value**	**Pr(>|z|)**
MT	-0.9	0.388
Treatment	-0.5	0.606
Time	2.0	0.047
Moisture	-0.3	0.761
MT : Time	1.9	0.063
MT : Treatment	0.9	0.329
Treatment : Time	1.1	0.408
MT : Treatment : Time	-1.7	0.09
**% Moisture**	**t_{DF}**	**p-value**
MT	$-2.2_{19.4}$	0.037
Time	$-3.8_{132.4}$	< 0.001
Treatment	$1.9_{19.0}$	0.078
MT : Time	$1.8_{131.5}$	0.079
MT : Treatment	$0.7_{19.2}$	0.476
Treatment : Time	$-0.2_{132.0}$	0.856
MT : Treatment : Time	$-0.1_{131.2}$	0.907

Table S4 (next page). Summary statistics for arbuscular mycorrhizal, ectomycorrhizal, saprotrophic, pathotrophic fungal biomass model results. MT represents mycorrhizal plot type, and treatment represents either control or logged plots. DenDF stands for denominator degrees of freedom. Numerator degrees of freedom were all 1. The pathotrophic fungal biomass model used the “glmmTMB” model to account for a large proportion of zeros in the dataset, and so we report z values and standard error. The time represents the number of months post harvest.

**Arbuscular mycorrhizal biomass (ug/g soil)**	**DenDF**	**F value**	**Pr(>F)**
MT	12	11.7	0.005
Treatment	12	9.1	0.011
Time	150	10.9	< 0.001
MT * Treatment	12	0.08	0.789
MT * Time	150	1.0	0.416
Time * Treatment	150	2.8	0.026
MT * Time * Treatment	150	1.0	0.407
**Ectomycorrhizal biomass (ug/g soil)**	**DenDF**	**F value**	**Pr(>F)**
MT	12	6.5	0.025
Treatment	12	1.9	0.20
Time	150	16.5	< 0.001
MT * Treatment	12	5.0	0.0456
MT * Time	150	0.9	0.456
Time * Treatment	150	3.5	0.010
MT * Time * Treatment	150	1.7	0.163
**Saprotrophic biomass (ug/g soil)**	**DenDF**	**F value**	**Pr(>F)**
MT	13	2.2	0.162
Treatment	162	0.1	0.73
Time	162	30.6	< 0.001
MT * Treatment	13	0.1	0.767
MT * Time	162	0.5	0.50
Time * Treatment	162	0.2	0.922
MT * Time * Treatment	162	1.7	0.143
**Pathotrophic biomass (ug/g soil)**	**Standard error**	**Z value**	**Pr(>|z|)**
MT	0.3	-1.8	0.069
Treatment	0.3	0.5	0.643
Time_3	0.4	-3.0	0.003
Time_7	0.3	-1.5	0.140
Time_15	0.3	0.4	0.670
Time_19	0.3	0.7	0.505

Table S5. Pairwise contrasts ("emmeans" function) between pre- and post-treatment control and harvested plots for AM and EcM biomass. Post-harvest time contrasts to pre-harvest data are in months since harvesting. Contrast estimates show pairwise differences in biomass (nmol per gram soil).

		**AM biomass**			**EcM biomass**		
Time	Season,Year	t ratio	p value	Contrast estimate	t ratio	p value	Contrast estimate
3	Spring, 2021	-1.6	0.078	-25280	-1.2	0.245	-4096
7	Fall, 2022	-3.1	0.002	-39482	-2.3	0.024	-7430
15	Spring, 2022	-1.6	0.054	-24299	-2.0	0.049	-6611
19	Fall, 2023	-1.3	0.19	-28246	-3.6	< 0.001	-11711

Table S6. Fungal community permanova results for the interaction between treatment and mycorrhizal legacy. We used the bray-curtis dissimilarity method and analyzed communities among years separately to account for separate sequencing runs.

**Year**	**R2**	**Pseudo F**	**P value**
2020	0.02	1.0	0.372
2021	0.01	1.1	0.102
2022	0.03	1.2	0.026

Table S7. Summary statistics for seedling relative growth rates from a linear mixed model. MST is the seedling mycorrhizal association, MT is the plot mycorrhizal legacy type, and condition is the presence or absence of an intact leader. Soil % moisture was measured for each seedling at one time point.

Term	DF	F value	Pr(>F)
**MST**	6.0	0.0	0.898
**MT**	6.0	0.1	0.746
**Condition**	1664.4	20.8	< 0.001
**MST : MT**	1661.0	1.1	0.571
**% moisture**	6932	0.1	0.74

References

Fitch, A., S. Goldsmith, E. Legge, T. D'Amato, K. Evans, and A. Kosiba. 2022. Exploring the role of mycorrhizal connections in forest regeneration and diversity. *New England Society of American Foresters*. https://nesaf.org/wp-content/uploads/2022/07/2022_July_NQ.pdf
